# Disparities in obesity-related cardiovascular-induced mortality trends in the U.S. of America over two decades

**DOI:** 10.3389/fcvm.2026.1736290

**Published:** 2026-03-20

**Authors:** Lingbo Huo, Lixing Ma, Yuyuan Wang, Haiyu Zhao

**Affiliations:** 1 Department of Hepatobiliary Surgery, Changzhi People’s Hospital, The Affiliated Hospital of Changzhi Medical College, Changzhi, Shanxi, China; 2 Department of Cardiology, Changzhi People’s Hospital, The Affiliated Hospital of Changzhi Medical College, Changzhi, Shanxi, China; 3 Department of Gerontology, Changzhi People’s Hospital, The Affiliated Hospital of Changzhi Medical College, Changzhi, Shanxi, China

**Keywords:** cardiovascular mortality, CDC WONDER, global disease burden, joinpoint regression, obesity

## Abstract

**Background:**

Cardiovascular disease (CVD) remains the leading cause of death in the U.S., and the growing prevalence of obesity has amplified this burden. Despite extensive research on their pathophysiologic interplay, long-term mortality trends among adults with both conditions remain insufficiently characterized.

**Methods:**

Using national death certificate data from the CDC WONDER database (1999–2020), we examined age-adjusted mortality rates (AAMRs) for CVD with obesity listed as a contributing cause among adults aged ≥25 years. Temporal trends were analyzed using joinpoint regression to estimate annual percent change (APC) and average annual percent change (AAPC) with 95% confidence intervals, stratified by sex, ethnicity, census region, state, and urbanization level.

**Results:**

From 1999 to 2020, the national AAMR increased steadily across all subgroups. Males had higher mortality and a steeper rise than females (AAPC 5.94% vs. 4.40%), with female rates accelerating after 2018. Non-Hispanic Black (NH Black) adults consistently experienced the highest mortality, while Hispanic and NH Black populations exhibited the sharpest recent increases. The South and Midwest regions showed the fastest growth (AAPC >5%), and nonmetropolitan areas surpassed metropolitan ones (AAPC 5.69% vs. 4.98%). Oklahoma displayed the most pronounced escalation nationwide (AAPC 10.52%).

**Conclusions:**

Over two decades, obesity-related CVD mortality has risen markedly and unevenly across demographic and geographic lines in the U.S. These findings highlight the intensifying intersection of cardiometabolic disease and social inequity, underscoring the urgent need for equity-centered prevention and investment in vulnerable communities.

## 1 Background

CVD remains the leading cause of mortality in the U.S., accounting for more deaths than any other chronic condition (1). Nearly 48.6% of U.S. adults are affected by some form of CVD, and the rising prevalence of obesity has further intensified this public health burden (2). Obesity, defined as a body mass index (BMI) ≥30 kg/m^2^, affects more than 42% of U.S. adults and represents a major modifiable risk factor for hypertension, dyslipidemia, type 2 diabetes, and coronary artery disease (3). The coexistence of obesity and CVD imposes a synergistic burden: obesity not only accelerates atherosclerotic progression but also contributes to adverse cardiovascular outcomes and premature mortality (4).

Prior research suggests that the association between obesity and CVD is mediated through complex metabolic, inflammatory, and neurohormonal pathways. Adipose tissue dysfunction promotes chronic low-grade inflammation, characterized by elevated levels of C-reactive protein, tumor necrosis factor-α, and interleukin-6, which are implicated in endothelial dysfunction and atherogenesis (5, 6). Obesity is also associated with insulin resistance and heightened sympathetic activity, both of which increase cardiac workload and contribute to structural remodeling (7, 8). However, the present study does not investigate these intermediate biological mechanisms; rather, it focuses on long-term, population-level mortality trends using nationally representative death certificate data.

Marked disparities in both obesity and CVD prevalence persist across demographic and geographic groups in the US. Non-Hispanic Black adults experience disproportionately higher rates of both conditions, reflecting structural inequities, socioeconomic barriers, and differential access to preventive care (9, 10). Although previous studies have described the pathophysiologic links between obesity and CVD, relatively few have systematically examined long-term mortality trends among individuals affected by both conditions. Therefore, this study aims to characterize disparities in CVD mortality among adults with obesity by sex, race, census region, state, and urbanization status over the past two decades. By delineating emerging epidemiologic patterns, this analysis seeks to inform more targeted and equitable public health interventions.

## 2 Methods

### 2.1 Data sources

The CDC's Wide-Ranging Online Data for Epidemiologic Research (WONDER) database was used to obtain death certificate data from 1999 to 2020 (11). CVD deaths were extracted using ICD-10 codes I00–I99 as the underlying cause of death, with obesity coded as a contributing cause (ICD-10: E66.0–E66.2, E66.8–E66.9) (12). CVD was operationalized as an aggregated underlying-cause ICD-10 category (I00–I99) in CDC WONDER; thus, findings represent group-level mortality surveillance rather than subtype-specific cardiovascular outcomes. Because the study used publicly available, de-identified data, Institutional Review Board approval was not required. This study is a retrospective population-based analysis using publicly available national mortality data. The study was conducted in accordance with the STROBE (Strengthening the Reporting of Observational Studies in Epidemiology) guidelines (13).

### 2.2 Study variables

We examined obesity contributing- CVD mortality by sex, ethnicity (NH White, NH Black, Hispanic, and NH other), census region (14), state, and urbanization status (metropolitan vs. nonmetropolitan, based on the 2013 NCHS Urban–Rural Classification Scheme) (15).

### 2.3 Statistical analysis

AAMRs per 100,000 population were calculated using the 2000 U.S. standard population. Temporal trends were assessed using joinpoint regression (Joinpoint Regression Program, National Cancer Institute), which identifies statistically significant inflection points and estimates APC and AAPC with 95% confidence intervals (16). Statistical significance was defined as *p* < 0.05. Statistically significant values are marked with an asterisk (*) in the results section. Analyses were stratified by geographic variables to evaluate disparities in mortality patterns.

## 3 Results

### 3.1 Trends by sex

**Figure 1:**
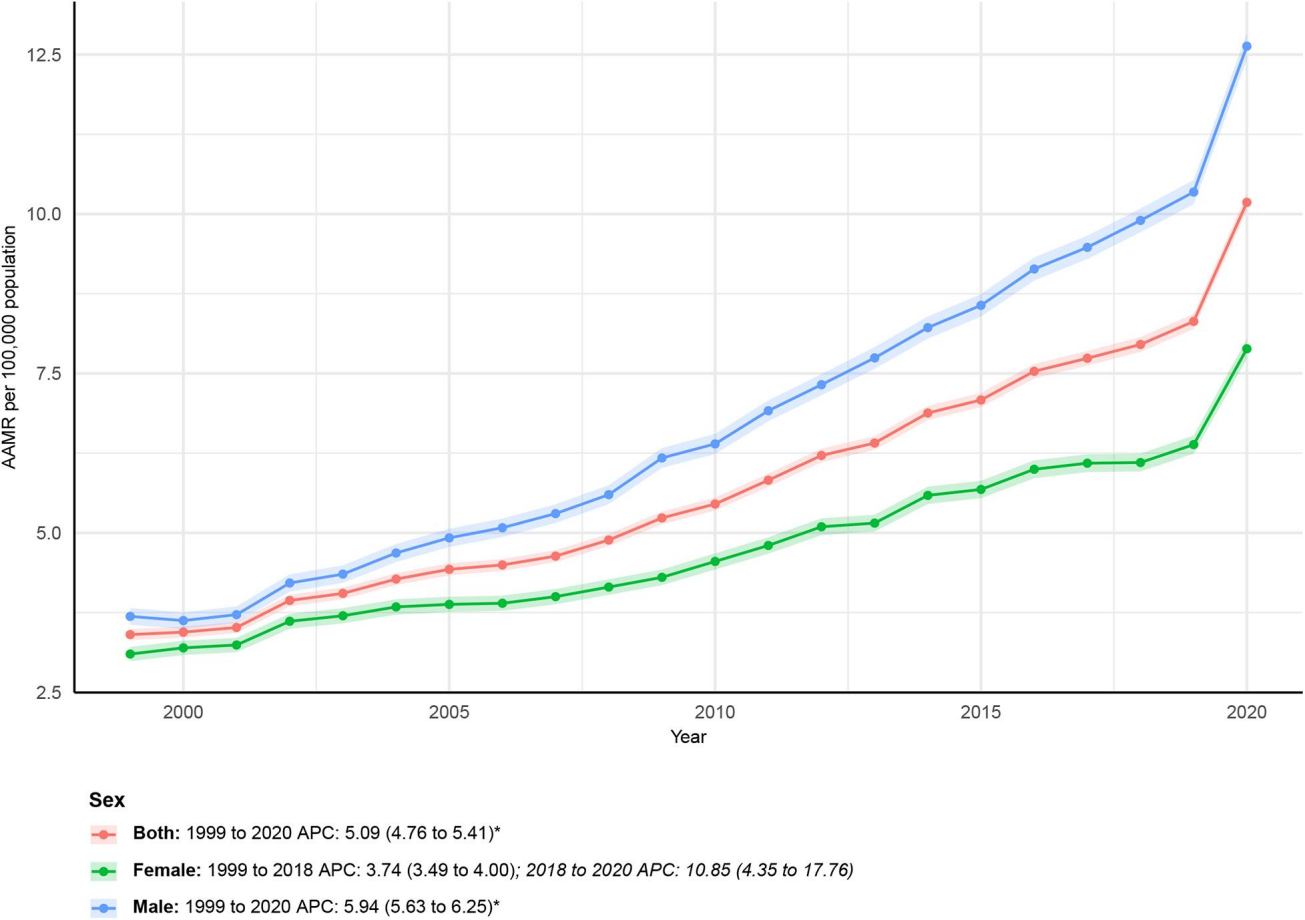
Sex-stratified deaths due to obesity-related cardiovascular-induced mortality: Age-adjusted mortality rates per 100,000 in the U.S., 1999–2020. APC, annual percent change.

From 1999 to 2020, the AAMR in males increased from 3.69 (95% CI: 3.56–3.82) in 1999 to 12.63 (95% CI: 12.42–12.84) in 2020, with an AAPC of 5.94 (95% CI: 5.63–6.25)*. The APC for males varied across time periods: 1999–2020: APC = 5.94 (95% CI: 5.63–6.25)*. In female patients, the AAMR also increased over the study period, from 3.10 (95% CI: 2.99–3.21) in 1999 to 7.89 (95% CI: 7.73–8.04) in 2020. The AAPC was 4.40 (95% CI: 3.80–5.00). The APC by time period for females was: 1999–2018: APC = 3.74 (95% CI: 3.49–4.00); 2018–2020: APC = 10.85 (95% CI: 4.35–17.76). All the above changes are visualized in Figure 1 and further detailed in Supplementary Table S1.

### 3.2 Trends by ethnicity

**Figure 2:**
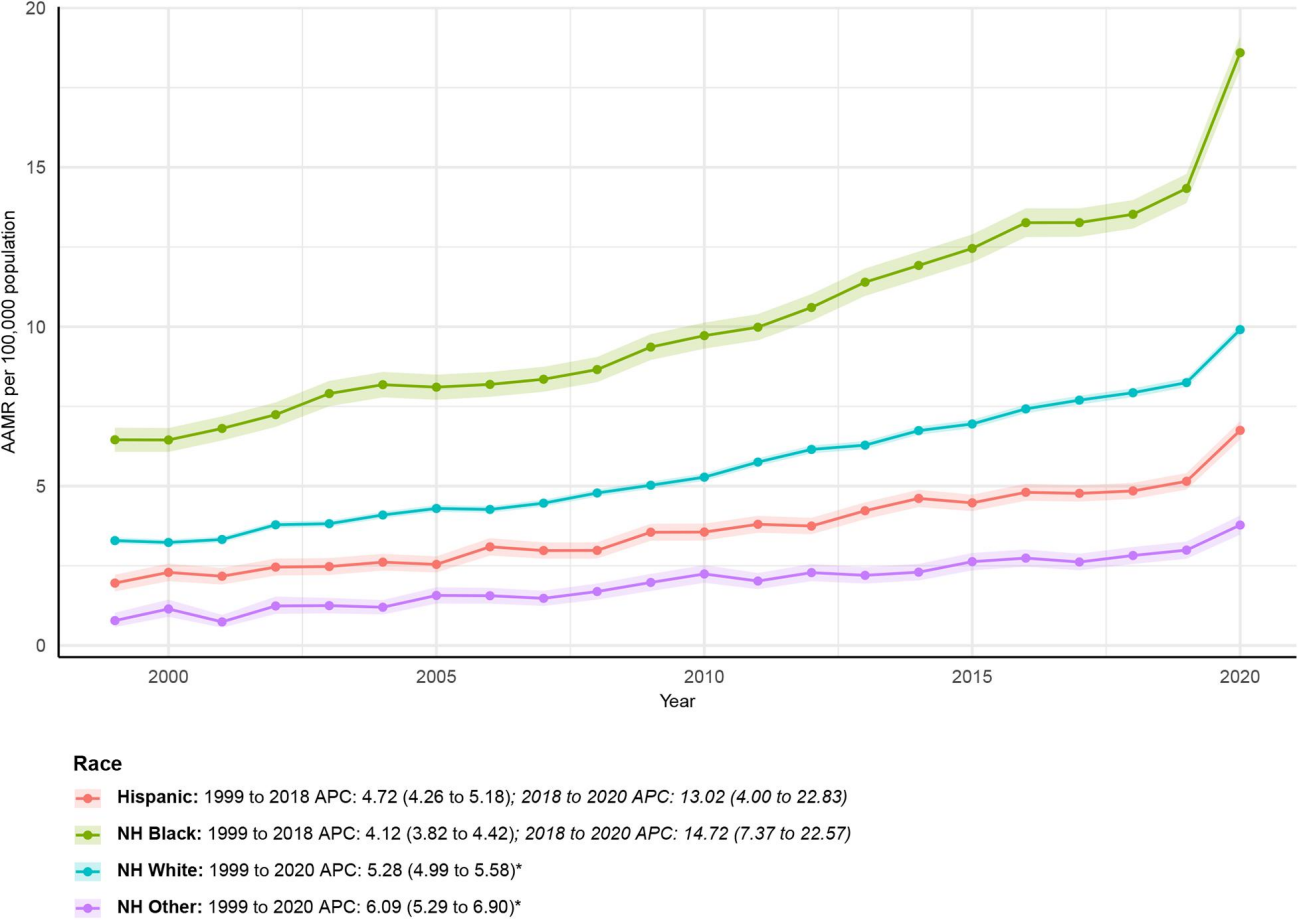
Ethnicity-stratified deaths due to obesity-related cardiovascular-induced mortality: Age-adjusted mortality rates per 100,000 in the U.S., 1999–2020. APC, annual percent change.

NH Other consistently had the lowest AAMR among racial groups, increasing from 0.78 (95% CI: 0.58–1.03) in 1999 to 3.78 (95% CI: 3.48–4.08) in 2020, with an AAPC of 6.09 (95% CI: 5.29–6.90)*. APC by time period for NH Other: 1999–2020: APC = 6.09 (95% CI: 5.29–6.90)*. Among NH White, AAMR decreased from 3.29 (95% CI: 3.19–3.39) in 1999 to 9.91 (95% CI: 9.75–10.06) in 2020. The AAPC was 5.28 (95% CI: 4.99–5.58)*. APC by time period: 1999–2020: APC = 5.28 (95% CI: 4.99–5.58)*. The AAMR for NH Black increased from 6.45 (95% CI: 6.07–6.83) in 1999 to 18.59 (95% CI: 18.08–19.11) in 2020. The AAPC was 5.09 (95% CI: 4.42–5.76)*. APC by time period: 1999–2018: APC = 4.12 (95% CI: 3.82–4.42); 2018–2020: APC = 14.72 (95% CI: 7.37–22.57). Among Hispanic patients, the AAMR increased from 1.96 (95% CI: 1.70–2.22) in 1999 to 6.75 (95% CI: 6.46–7.04) in 2020. The AAPC was 5.48 (95% CI: 4.62–6.36)*. APC by time period: 1999–2018: APC = 4.72 (95% CI: 4.26–5.18); 2018–2020: APC = 13.02 (95% CI: 4.00–22.83). See Figure 2 and Supplementary Table S1 for detailed trends for all racial groups.

### 3.3 Trends by census regions

**Figure 3:**
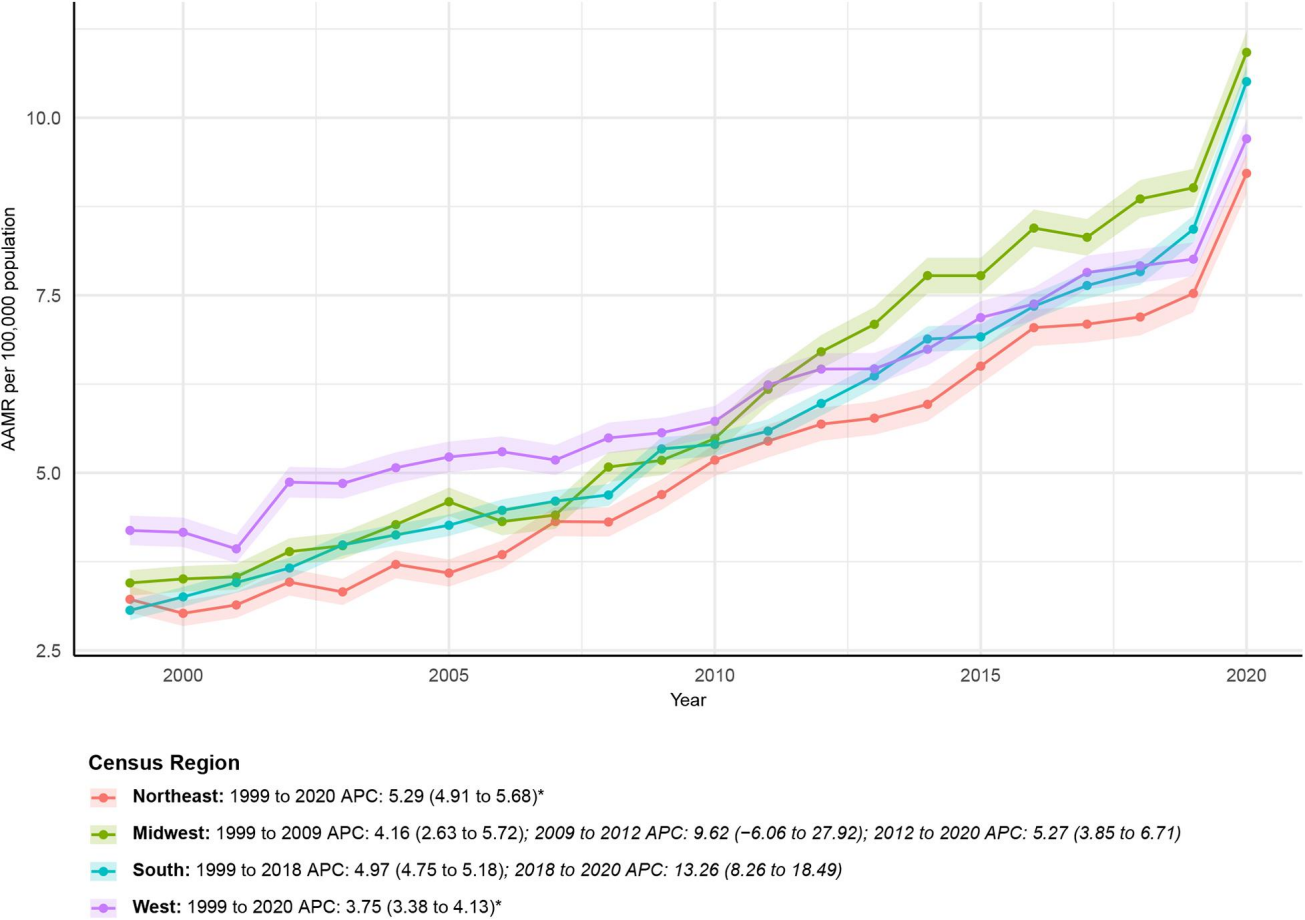
Census regions-stratified deaths due to obesity-related cardiovascular-induced mortality: Age-adjusted mortality rates per 100,000 in the U.S., 1999–2020. APC, annual percent change.

All census regions showed an increase in AAMR over time. The AAMR for Midwest increased from 3.45 (95% CI: 3.27–3.63) in 1999 to 10.92 (95% CI: 10.63–11.21) in 2020. The AAPC was 5.35 (95% CI: 3.09–7.66)*. APC by time period: 1999–2009: APC = 4.16 (95% CI: 2.63–5.72); 2009–2012: APC = 9.62 (95% CI: −6.06–27.92); 2012–2020: APC = 5.27 (95% CI: 3.85–6.71). Among Northeast, the AAMR increased from 3.22 (95% CI: 3.03–3.40) in 1999 to 9.22 (95% CI: 8.93–9.51) in 2020. The AAPC was 5.29 (95% CI: 4.91–5.68)*. APC by time period: 1999–2020: APC = 5.29 (95% CI: 4.91–5.68)*. For South, the AAMR also increased over the study period, from 3.06 (95% CI: 2.93–3.20) in 1999 to 10.51 (95% CI: 10.30–10.72) in 2020. The AAPC was 5.73 (95% CI: 5.27–6.19)*. The APC by time period for South was: 1999–2018: APC = 4.97 (95% CI: 4.75–5.18); 2018–2020: APC = 13.26 (95% CI: 8.26–18.49). Among West, the AAMR increased from 4.19 (95% CI: 3.98–4.40) in 1999 to 9.70 (95% CI: 9.45–9.96) in 2020. The AAPC was 3.75 (95% CI: 3.38–4.13)*. APC by time period: 1999–2020: APC = 3.75 (95% CI: 3.38–4.13)*. Regional differences are illustrated in Figure 3 and further detailed in Supplementary Table S1.

### 3.4 Trends by urbanization

**Figure 4:**
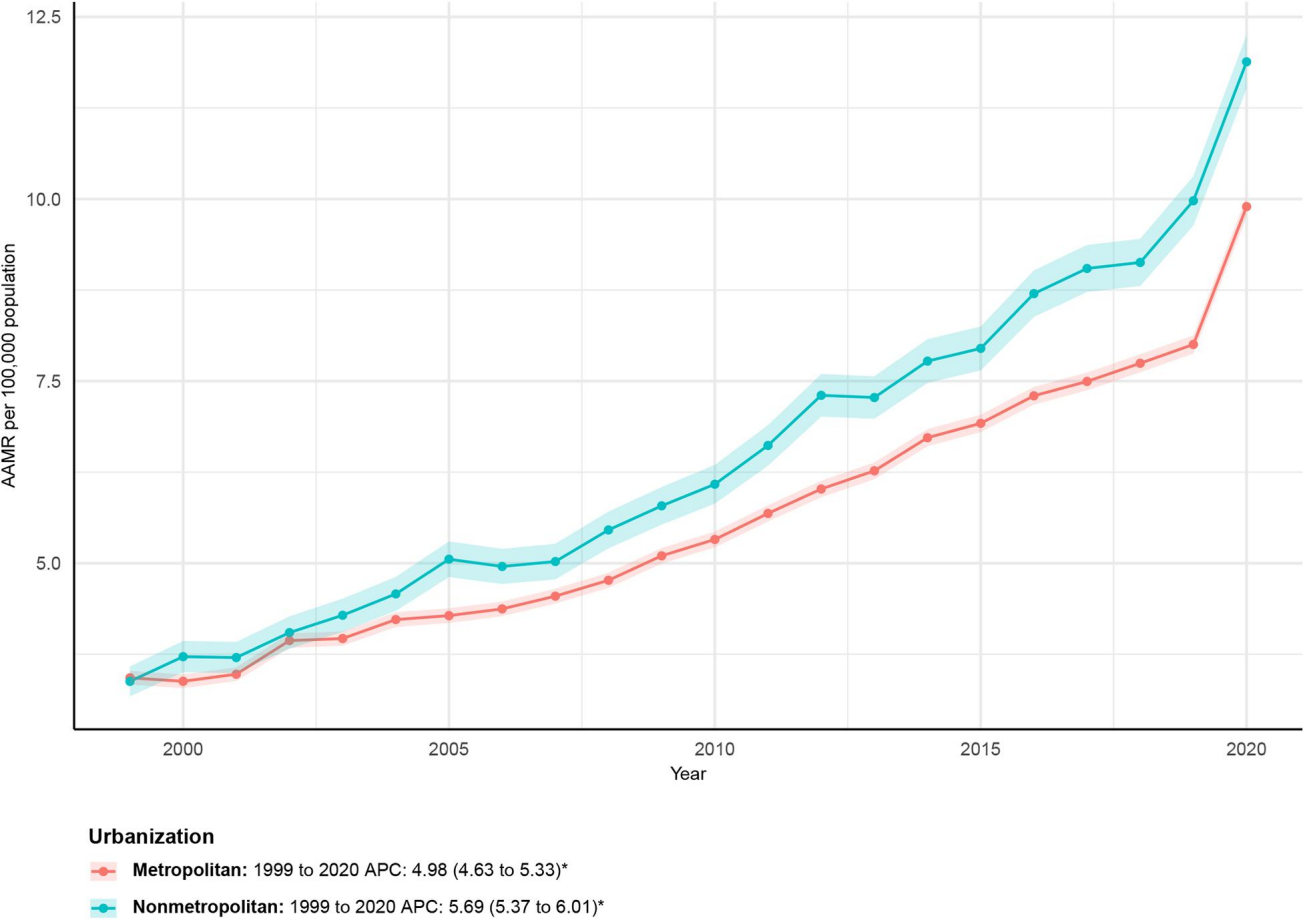
Urbanization-stratified deaths due to obesity-related cardiovascular-induced mortality: Age-adjusted mortality rates per 100,000 in the U.S., 1999–2020. APC, annual percent change.

From 1999 to 2020, the AAMR in metropolitan areas increased from 3.43 (95% CI: 3.33–3.52) in 1999 to 9.90 (95% CI: 9.76–10.03) in 2020, with an AAPC of 4.98 (95% CI: 4.63–5.33)*. APC for metropolitan areas: 1999–2020: APC = 4.98 (95% CI: 4.63–5.33)*. In nonmetropolitan areas, the AAMR also increased, from 3.38 (95% CI: 3.17–3.58) in 1999 to 11.88 (95% CI: 11.52–12.25) in 2020. The AAPC was 5.69 (95% CI: 5.37–6.01)*. APC by time period: 1999–2020: APC = 5.69 (95% CI: 5.37–6.01)*. See Figure 4 and Supplementary Table S1 for details.

### 3.5 Distribution of state-level mortality and trends in the U.S

**Figure 5:**
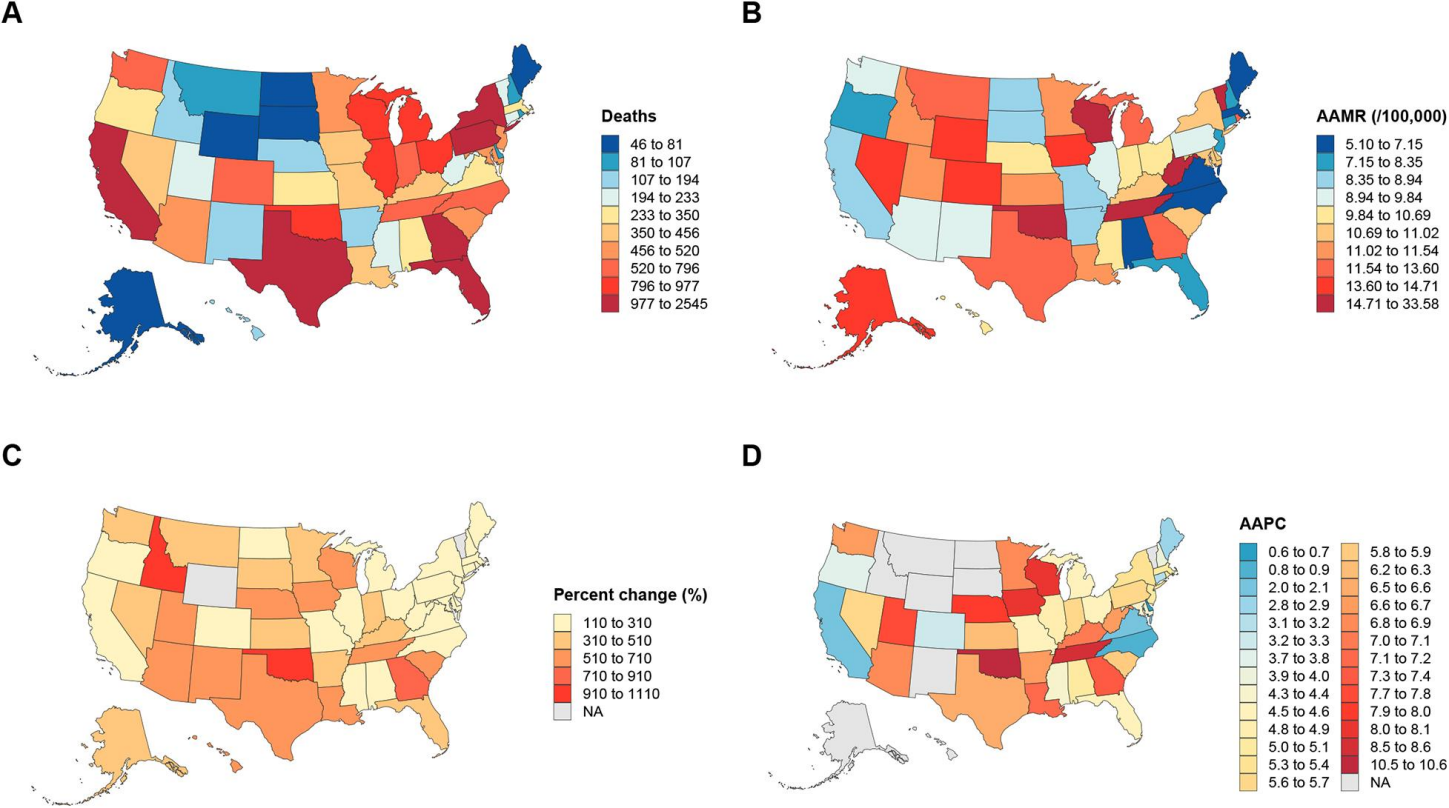
Distribution of state-level mortality and trends in the U.S. **(A)** State-level total deaths in 2020. **(B)** AAMR in 2020. **(C)** Percentage change in deaths from 1999 to 2020. **(D)** AAPC from 1999 to 2020. AAMR, age-adjusted mortality rate; AAPC, average annual percent change.

In the spatial analysis, we mapped state-level total deaths (Figure 5A) and AAMR (Figure 5B) in 2020, as well as the percentage change in deaths (Figure 5C) and AAPC (Figure 5D) from 1999 to 2020. State-level distributions of deaths and AAMR were displayed using discrete classification with fixed legends, ensuring comparability across states. While some states reported the highest absolute number of deaths in 2020, their corresponding AAMR values were not always the highest, underscoring the influence of population size on absolute mortality counts (Supplementary Table S1). In addition, Oklahoma exhibited one of the steepest increases in age-adjusted mortality rate (AAMR) nationwide, rising from 3.01 (95% CI: 2.33–3.82) in 1999 to 27.55 (95% CI: 25.58–29.52) in 2020, with an AAPC of 10.52% (95% CI: 7.87–13.25). The percentage change in deaths over 1999–2020 was visualized with a warm color scale, highlighting substantial heterogeneity in growth magnitude across states. Most states experienced an increase, but the extent of change varied considerably. Finally, the distribution of AAPC was represented on a blue-to-red gradient, with positive values indicating an increase. Most states exhibited positive AAPC, consistent with a long-term upward trend (Supplementary Table S1).

## 4 Discussion

From 1999 to 2020, AAMR increased substantially across all examined sex, racial/ethnic, regional, urbanization, and state subgroups. The consistent upward trajectory, with notable acceleration after 2018, indicates an expanding mortality burden rather than short-term fluctuation.

Males consistently demonstrated higher AAMR than females, with a steeper overall increase (AAPC 5.94% vs. 4.40%). This persistent excess male mortality parallels national patterns in cardiometabolic and cardiovascular disease, where male mortality is typically 1.3–1.8 times higher than female mortality (17, 18). The male-to-female ratio remained stable over time, indicating sustained disparity.

In contrast, females experienced a marked acceleration after 2018 (APC 10.85%), representing a shift from prior gradual growth. National data documenting rising obesity, metabolic dysfunction, and psychosocial stressors among women during the late 2010s temporally align with this inflection (19, 20). Although causal inference cannot be drawn from aggregate data, the timing suggests evolving risk dynamics.

NH Black individuals had the highest AAMR throughout the study period, whereas NH Other groups had the lowest rates. The magnitude of disparity is consistent with prior national analyses demonstrating disproportionate chronic disease mortality among NH Black populations (21, 22). Notably, acceleration after 2018 was most pronounced among Hispanic and NH Black populations, indicating widening recent disparities.

These patterns align with extensive evidence linking structural inequities—including differential access to preventive care, higher comorbidity burden, and neighborhood-level socioeconomic disadvantage—to racialized mortality gradients (23, 24). While mechanisms cannot be directly assessed in this ecological framework, the consistency with established literature supports the robustness of these observations.

Geographic heterogeneity was substantial. The South and Midwest exhibited the highest AAPC values, with the South showing a sharp post-2018 acceleration (APC 13.26%) exceeding national growth rates. These regions have historically carried higher burdens of obesity, diabetes, and limited healthcare access (25, 26), suggesting persistence and intensification of preexisting vulnerabilities.

Mortality increased across all urbanization levels; however, nonmetropolitan areas experienced faster growth than metropolitan areas (AAPC 5.69% vs. 4.98%). This widening urban–rural gap mirrors national reports of accelerated mortality growth in rural communities across multiple causes (27, 28). Structural factors—including aging populations, hospital closures, and limited specialty care—likely contribute to this divergence.

Oklahoma exhibited one of the steepest increases nationwide, with an AAPC nearly fourfold higher than the national average. Public health surveillance consistently reports higher prevalence of obesity, smoking, uninsured status, and cardiometabolic disease in Oklahoma compared with national averages (29–31). The convergence of elevated risk factor burden and accelerated mortality growth highlights substantial state-level vulnerability. Although causation cannot be inferred, these findings underscore the importance of locally tailored interventions.

The uniform upward trend across demographic and geographic strata suggests systemic drivers rather than isolated population effects. The post-2018 acceleration across multiple subgroups warrants focused investigation, including potential contributions from healthcare disruptions, shifts in preventive care utilization, and early indirect effects of the COVID-19 pandemic (32). Future studies integrating individual-level risk factor and healthcare utilization data are needed to clarify causal pathways.

## Limitations

This study has several limitations. First, the analysis is ecological and based on aggregated mortality data; therefore, causal inferences at the individual level cannot be established. Second, mortality estimates rely on death certificate data, which may be subject to misclassification and potential temporal changes in coding practices. Third, the absence of individual-level clinical, socioeconomic, and behavioral information precludes adjustment for important confounders and limits mechanistic interpretation of the observed disparities. Fourth, while joinpoint regression identifies statistically significant inflection points in temporal trends, it does not provide insight into the underlying drivers of these changes. Fifth, data were available only through 2020; thus, more recent trends require continued surveillance. Additionally, because ICD-10 codes I00–I99 encompass a broad spectrum of cardiovascular conditions with heterogeneous etiologies, the observed trends likely reflect varying contributions from different CVD subtypes. Our analysis was not designed to disentangle subtype-specific mechanisms or attribute changes to particular cardiovascular diagnoses. Despite these limitations, the use of nationally representative data with standardized methodology over a prolonged period offers robust and valuable insight into long-term mortality patterns in the U.S.

## Conclusion

Between 1999 and 2020, AAMR increased markedly across the U.S., with persistent and in some cases widening disparities by sex, ethnicity, region, urbanization, and state. The acceleration observed after 2018 suggests intensifying underlying risk dynamics. These findings highlight the need for equity-focused prevention strategies and sustained investment in healthcare access and chronic disease mitigation. Without targeted structural interventions, existing mortality gradients are likely to persist or expand.

## Data Availability

The original contributions presented in the study are included in the article/Supplementary Material, further inquiries can be directed to the corresponding author.
